# Efficacy of Wii-Fit on Static and Dynamic Balance in Community Dwelling Older Veterans: A Randomized Controlled Pilot Trial

**DOI:** 10.1155/2017/4653635

**Published:** 2017-02-05

**Authors:** Kalpana P. Padala, Prasad R. Padala, Shelly Y. Lensing, Richard A. Dennis, Melinda M. Bopp, Christopher M. Parkes, Mark K. Garrison, Patricia M. Dubbert, Paula K. Roberson, Dennis H. Sullivan

**Affiliations:** ^1^Geriatric Research Education and Clinical Center, Central Arkansas Veterans Healthcare System, Little Rock, AR, USA; ^2^Department of Geriatrics, University of Arkansas for Medical Sciences, Little Rock, AR, USA; ^3^Department of Psychiatry, University of Arkansas for Medical Sciences, Little Rock, AR, USA; ^4^Department of Biostatistics, University of Arkansas for Medical Sciences, Little Rock, AR, USA; ^5^Department of Physical Therapy, University of Central Arkansas, Conway, AR, USA

## Abstract

*Background/Objectives*. Balance problems are well-established modifiable risk factors for falls, which are common in older adults. The objective of this study was to establish the efficacy of a Wii-Fit interactive video-game-led physical exercise program to improve balance in older Veterans.* Methods*. A prospective randomized controlled parallel-group trial was conducted at Veterans Affairs Medical Center. Thirty community dwelling Veterans aged 68 (±6.7) years were randomized to either the exercise or control groups. The exercise group performed Wii-Fit program while the control group performed a computer-based cognitive program for 45 minutes, three days per week for 8-weeks. The primary (Berg Balance Scale (BBS)) and secondary outcomes (fear of falling, physical activity enjoyment, and quality of life) were measured at baseline, 4 weeks, and 8 weeks.* Results*. Of 30 randomized subjects, 27 completed all aspects of the study protocol. There were no study-related adverse events. Intent-to-treat analysis showed a significantly greater improvement in BBS in the exercise group (6.0; 95% CI, 5.1–6.9) compared to the control group (0.5; 95% CI, −0.3–1.3) at 8 weeks (average intergroup difference (95% CI), 5.5 (4.3–6.7),* p* < 0.001) after adjusting for baseline.* Conclusion*. This study establishes that the Wii-Fit exercise program is efficacious in improving balance in community dwelling older Veterans. This trial is registered with ClinicalTrials.gov Identifier NCT02190045.

## 1. Introduction

Gait and balance problems increase with age, making older adults vulnerable to falls. Community dwelling adults, aged 65 years or older, have a 22% yearly incidence of falls [1]. Falls are associated with increased morbidity and mortality [2, 3]. For example, falls are the preeminent cause of hip fractures in older adults, which in turn are associated with decreased mobility, quality of life, and premature death. Among older adults hospitalized as a result of a fall, only 50% are alive one year later [4]. Although there are numerous intrinsic and extrinsic risk factors for falls, gait and balance problems are the most consistent predictors of future falls [5]. Balance is a complex issue with components of static and dynamic stability, sensory integration, and reactive postural control [6]. Some of these components can be improved by interventions making balance a modifiable risk factor for falls [7, 8]. The current study primarily targets static and dynamic stability.

Exercise improves gait and balance in the elderly. A Cochrane review found statistically significant improvement in balance following exercise interventions compared to usual activity [8]. Another review of 54 exercise studies in older adults concluded that interventions with challenging balance training and high dose exercise (≥2 hrs/wk) had the greatest impact on preventing falls [9]. Even low intensity exercises improve balance and gait, particularly in deconditioned elders at high risk of falling [10]. Furthermore, the benefits of exercise on reducing falls and injuries accumulate over time suggesting a need for adopting exercise as a lifestyle [11]. Despite the many proven benefits of exercise, older adults in the USA remain sedentary and show declines in moderate and vigorous physical activity with increasing age. Among adults aged 65 to 74 years, only 34% of men and 17% of women expend more than 2000 kcal per week in exercise [12]. Thus, a novel approach to increase the time engaged by older adults in exercise by making the exercises fun and safe would be valuable.

Exergames are interactive video games involving physical activity or exertion tracked as body movement [13]. The technology may enable sedentary older adults to safely add exercise to their lifestyle. The Nintendo Wii-Fit® exergame provides aerobics, strength, and balance training using a balance board that senses shifts in weight [14]. This gaming system is widely available and affordable and has the potential for self-directed home use by older adults. Even though the Wii-Fit program has been found to be safe and feasible in older adults, the efficacy of the program has not been well-established. Thus, the primary objective of the current study was to test the efficacy of an 8-week Wii-Fit program for improving balance in older Veterans with balance problems. The secondary objectives were to explore the effects of the exergames on fear of falling, exercise enjoyment, and quality of life.

## 2. Methods

### 2.1. Study Design and Participants

This pilot study was an 8-week, prospective randomized controlled parallel-group trial comparing the effects on measures of balance in the exercise group (Wii-Fit program) to that in the control group (Brain-Fitness program). The study was conducted at Department of Veterans Affairs Medical Center. The protocol was approved by the Institutional Review Board of the Central Arkansas Veterans Healthcare System. Thirty subjects were recruited to inform sample size calculations for a larger study. After responding to advertisements, recruits were prescreened by medical records review to determine eligibility. Community dwelling adults aged ≥ 60 years were included in the study. Subjects using wheel chairs or walkers for mobility, having absolute contraindications to exercise per American College of Sports Medicine guidelines, and those with medical conditions that in the opinion of the study physician were likely to compromise safe study participation were excluded [15].

### 2.2. Study Procedure

Subjects who cleared the prescreening were invited for the baseline visit during which they provided a written informed consent. At this visit, eligibility was further assessed with Berg Balance Scale (BBS) and Mini Mental State Exam (MMSE). Those that scored BBS ≤ 52 and MMSE ≥ 24 were included in the study. The cut-off score of 52 on BBS was selected in an effort to avoid ceiling effects and to include those with mild-to-moderate balance problems. Demographics and anthropometric data were collected at the baseline visit. Eligible subjects were trained to use Wii-Fit and Brain-Fitness programs over one to two sessions to ensure they were willing and capable of safe participation in either program. Subjects were randomized using a randomized block design to the exercise (*n* = 15) and control groups (*n* = 15) using sealed envelopes prepared by the statistician. Research assistants monitored participation in both groups for exercise fidelity, safety, and compliance. Participation time and activities performed were recorded. Subjects were allowed to choose the activities performed for Wii-Fit or Brain-Fitness though research assistants encouraged progression to higher level and inclusion of activities from each category, described below, at each session.

### 2.3. Exercise Group

Subjects trained for 45 minutes three days per week for 8 weeks in a well-lit exercise suite in a VA Medical Center. The room was set such that sufficient space was available for exercises free of any obstacles or fall hazards. The Wii-Fit group performed exercises from five categories: Yoga, Strength Training, Aerobics, Balance Games, and Training Plus which includes more complex exercise tasks. Balance exercises involved static and dynamic postural control (Half Moon, Torso Twist, and Deep Breathing), lateral weight shifting (Ski Slalom, Penguin Slide, and Tight Rope Walking), multidirectional balance (Table Tilt, Balance Bubble), and multidirectional balance with a cognitive component (Perfect 10). Subjects tracked their activity and progress using an individualized icon (“Mii,” virtual avatar). Each session included a warm-up, exercise, and cool-down phase. During the warm-up and cool-down phases, subjects walked for five minutes at a self-selected comfortable pace using the program's “basic walk” activity. The exercise phase was designed to be patient centric. Instead of doing exactly the same exercises, subjects were encouraged to choose one or more exercises from every Wii-Fit category during each session. Research assistants were in the exercise room during the entire session maintaining visual contact and being readily available if necessary.

### 2.4. Control Group

Subjects performed cognitive exercises using a commercially available computer program, Brain-Fitness (HAPPYneuron Inc., Lyon, France) [16, 17] for 45 minutes three days per week for 8 weeks. The control group performed the cognitive exercises in a well-lit room at the VA Medical Center at the same frequency to ensure equal contact of all subjects with the research team. The program focused on five cognitive domains: memory (Entangled Figures, Turn Around and Around, and Money Time), language (Split Words, Right Words, Squeaky Mouse, Secret Files, and Dance with the Fireflies), attention (Split Words, Right Words, and Towers of Hanoi), executive function (American in Paris, Objects Where Are You), and visual and spatial domain (Around the World in 80 Trips).

### 2.5. Primary Outcomes Measures

All outcomes were measured at baseline and four and eight weeks after intervention initiation. The primary efficacy outcome was the Berg Balance Scale (BBS), which assesses balance impairments in older adults and is a good measure of static and dynamic stability [18]. It consists of 14 tasks performed in a standardized order with each task scored on a five-point scale according to quality or time ranging from “0” (lowest level of function) to “4” (highest level). The maximum score is 56. BBS has an excellent interrater reliability (0.98) [18]. A change of four points is considered the minimally detectable change for community dwelling older adults that ambulate without an assistive device [19].

### 2.6. Secondary Outcome Measures

The secondary outcomes included Activities-Specific Balance Confidence (ABC) scale, Physical Activity Enjoyment Scale (PACES), Modified Mini Mental State Exam (3MS), Rand Short Form 36 (SF-36), and incidence of adverse events. The ABC scale measures fear of falling and self-confidence to maintain balance [20]. Subjects rate their confidence in maintaining balance while engaging in 16 nonhazardous activities of daily living. Lower scores indicate greater fear of falling. The test-retest reliability is 0.92 with Cronbach's alpha of 0.96 [20]. PACES scale (8-item version) assesses perceived enjoyment of exercise [21, 22]. PACES score ranges from 8 to 56. Higher scores signify greater enjoyment. It has good validity and internal consistency (0.93) [21, 22].

3MS is a global screen for cognitive function that covers orientation to time and place, registration, recall, simple language, and construction. Test-retest reliability ranges from 0.91 to 0.93 [23]. The total score places the individual on a well-accepted scale of cognitive function. Different versions were used at each testing occasion to minimize practice effects. An MMSE score derived from 3MS was used in the inclusion criteria of the study. The Rand Short Form-36 is a generic measure of quality of life (QOL). The two components (mental and physical) and the physical function scores were utilized. Each component ranges from 0 to 100, with higher values indicating better QOL. It has good interrater reliability of 0.76–0.96 [24]. Adverse events (near falls, falls, dizziness, joint, muscle or chest pain, and shortness of breath) were monitored via medical records review before each session and by subject query during the sessions.

### 2.7. Statistical Analyses

Subject demographics and baseline characteristics were compared using* t*-tests or nonparametric Wilcoxon rank sum tests (continuous data) and Fisher's exact tests (categorical data). Changes from baseline at 4 and 8 weeks in primary and secondary outcomes were analyzed using repeated measures mixed model analyses of covariance. Group (exercise and control) and time (4 and 8 weeks) as well as the interaction between the two were independent variables; the dependent variable's baseline measure was included as a covariate. In post hoc analyses, groups were compared in terms of the changes from baseline at 4 and 8 weeks using* t*-tests derived from model-based contrasts. Additionally, comparisons were made to separately assess improvements within each group. A sensitivity analysis to investigate bias due to missing data for subjects (*n* = 3) who dropped out of the study was performed by carrying forward baseline values to conservatively impute missing data as no change; we also investigated the impact of including MMSE and SF-36 Physical and Mental summary scores as covariates in the sensitivity analysis. Two-sided* p *values less than 0.05 indicated statistical significance. Data were analyzed using SAS Enterprise Guide v5.1 (SAS, Cary, SC).

## 3. Results 

**Figure 1:**
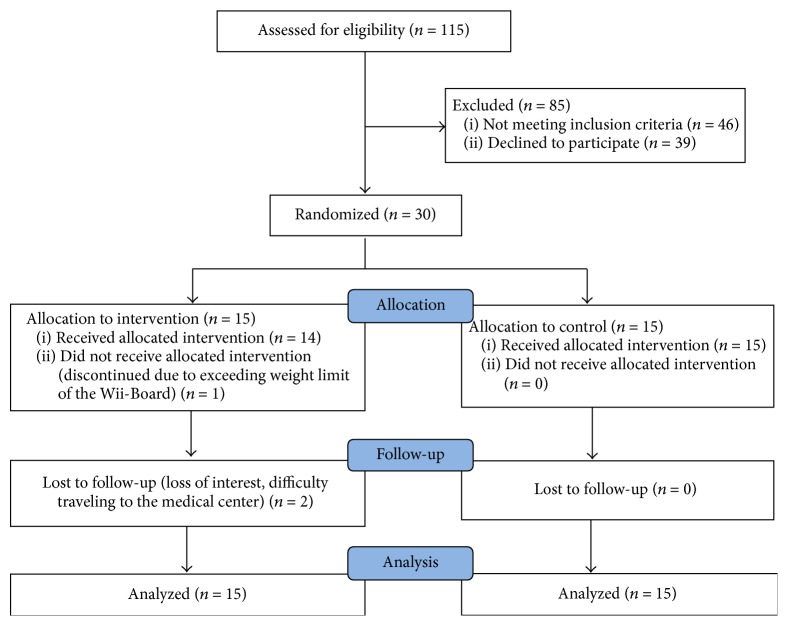
Screening, enrollment, and participation.

Figure 1 depicts the screening, enrollment, and participation process. A total of 115 subjects were prescreened (record review) of which 79 subjects were found eligible for in-person screening. These subjects were invited to come for the baseline visit. Of the 40 subjects evaluated at the baseline visit, 30 met the full eligibility criteria and were randomized. Three subjects dropped out from the exercise group. One was withdrawn after randomization as his weight exceeded the weight limit for the Wii-Board. The other two subjects dropped out of the study due to loss of interest and difficulties traveling to the medical center, respectively. The demographic characteristics of the study sample are presented in Table 1. Twenty-six out of 30 subjects were male, which is representative of the Veteran population age ≥ 60 years. There were no significant differences between the two groups with regard to age, race, gender, medical comorbidities, and medications (Table 1). As shown in Table 2, there were no significant differences at baseline between the two groups with regard to primary or secondary outcome measures except for MMSE. All study visits were attended by the 27 subjects who completed the study (100% adherence). Subjects did not experience any study-related adverse events. At no time during the study did any subject need any physical help from the research assistants. Subjects were able to operate the video game remote and perform the exercises on their own. All subjects completed the stipulated exercise time at each session.

### 3.1. Program Preferences

Balance Games were the most accessed exercises in the exercise group (Ski Jump (median 11 of 24 visits), followed by Bubble Balance, Penguin Slide, and Table Tilt (median of 9, 8, and 7.5 visits, respectively)). Simple Yoga poses (Deep Breathing, Half Moon, and Warrior), Strength Training (Torso Twist, Triceps Extension), Training Plus (Perfect 10, Obstacle Course, Island Cycling, and Segway Circuit), and Balance Games (Soccer Heading, Ski Slalom, Tight Rope Walking, and Lotus Focus) were chosen at least once by at least 50% of the participants. Some of the outcomes could depend on the game preferences, the details of which are included in Table 4. Subjects in the control group accessed all the categories frequently (median ≥ 20 of 24 visits). Brain games involving attention and language (Split Words, Towers of Hanoi, and Right Word) and executive function (American in Paris) were the most accessed.

### 3.2. Outcome Measures

**Figure 2:**
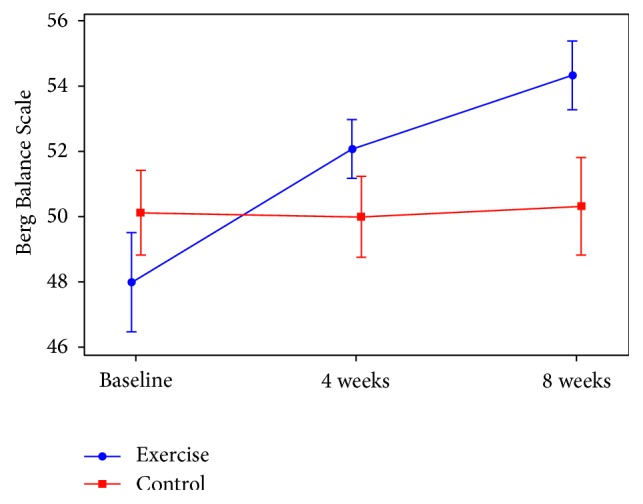
Berg Balance Scale over time in exercise and control groups (observed mean ± 2SE).

For the subjects who completed the study (*N* = 27), there was a significant group-by-time interaction for the primary outcome measure BBS (*p* < 0.001, Figure 2). Post hoc analysis showed that the change in BBS from baseline for the exercise group significantly differed from that in the control group at both 4 and 8 weeks. After adjusting for baseline BBS score, the average between-group differences (95% CI) were 3.6 (2.3–4.8; *p* < 0.001) at 4 weeks and 5.5 (4.3–6.7; *p* < 0.001) at 8 weeks. Within-group analysis showed significant improvement in BBS in the exercise group at 4 weeks (3.7 [2.8–4.6], *p* < 0.001) and at 8 weeks (6.0 [5.1–6.9], *p* < 0.001) but no improvement in the control group at 4 weeks (0.2 [−0.6–0.9], *p* = 0.70) or at 8 weeks (0.5 [−0.3–1.3], *p* = 0.22). All subjects in the exercise group who completed the study demonstrated a clinically relevant change of greater than or equal to four on the BBS at 8 weeks [19]. Additionally, when the missing outcomes data for the three subjects in the exercise group were imputed as no change in a sensitivity analysis (intent-to-treat approach), the effect of exercise was decreased but still statistically significant: the average between-group differences (95% CI) were 2.7 (1.2–4.2, *p* = 0.001) at 4 weeks and 4.1 (2.6–5.7, *p* < 0.001) at 8 weeks. Adjusting for MMSE and SF-36 Physical and Mental summary scores resulted in between-group differences that were only slightly (<10%) larger, and these covariates were nonsignificant (all *p* > 0.15, results not shown).

There was a significant average improvement (95% CI) within the exercise group at 8 weeks on the secondary outcome measure, ABC scale (5.9 [0.1–11.6], *p* = 0.046) (Table 3). Analysis of exercise enjoyment at the end of 8 weeks was conducted in the Wii-Fit arm. The average score on the PACES was 49.2 (±7.4) on a 56-point scale. 83% of the subjects rated Wii-Fit to be high (6 or 7) on the measure of pleasure. A similarly high percentage rated Wii-Fit to be fun (75%), pleasant (75%), invigorating (67%), gratifying (83%), exhilarating (83%), stimulating (92%), and refreshing (92%). There were significant within-group improvements in the exercise group at 4 weeks and at 8 weeks and in control group at 4 weeks for both 3MS and MMSE (*p* < 0.05 for all comparisons, Table 3). There was a significant within-group improvement in SF-36 Physical function scale at 4 weeks in the exercise group (9.2 [0.5–17.9], *p* = 0.039) but not at 8 weeks. However, the group-by-time effect was nonsignificant for these variables. There were no significant group-by-time interactions or between-group differences for any of the other secondary outcomes.

## 4. Discussion

The study sought to establish the efficacy of Wii-Fit exercise for improving balance in older adults with mild-to-moderate balance problems. As in prior studies, participants in this study experienced no adverse events, indicating that older adults with comorbidities can safely complete the program. The study also verified that Wii-Fit is feasible to use for training since the adherence rate was high and participants were able to exercise independently. Efficacy was demonstrated for the primary outcome, BBS. The average BBS change was six points and all subjects improved by at least four points at 8 weeks, a clinically relevant change observed in a similar community dwelling population [19]. Change in BBS can be influenced by baseline scores [25], but the baseline difference between groups in the current study was modest, neither statistically nor clinically significant (*p* = 0.08). Additionally, our analyses adjusted for baseline scores to further validate the effects of the program on balance. The study was not a priori powered to detect differences in secondary outcomes.

Balance-board-based exercises have been used to improve balance [26]. Wii-Fit is a balance board exercise system that has been tested as an exercise intervention using various study designs with mixed results. Noncontrolled trials have shown that Wii-Fit is safe and feasible for older adults [27–30]. Wii-Fit has been safely used in a variety of patient populations such as stroke, Parkinson's disease, and Alzheimer's disease. RCTs have never associated Wii-Fit with harm but multiple studies have failed to find differences between the intervention and control groups for multiple outcomes including balance, where balance was either a primary or secondary outcome measure [31–35].

As described in recent reviews, 35 prior studies examined the use of Wii-Fit or other balance-board-based exercise games to improve balance or motor function [26, 36]. Of these 35 articles, 21 (60%) were RCTs [26, 36] and 15 (43%) were RCT using Wii-Fit [26, 36]. Sixteen (76%) of the 21 RCTs examined balance as an outcome; six (38%) of these studies found significant between-group differences in balance [26, 36]. However, all six of these studies had important methodologic limitations [37–42]. In all but one study (83%), subjects in the intervention group had more contact with the research staff than did the control subjects who were mainly seen to complete baseline and outcome evaluations [37–40, 42]. Intent-to-treat analysis was not utilized in three of the six studies (50%) despite having dropout rates ranging from 6% to 39% [38, 39, 41]. One study (17%) did not control for baseline differences in the primary outcome measure of balance even though these differences were statistically significant [41]. Only one of the six RCTs that reported significant between-group differences used Wii-Fit as an exercise intervention [40]. In this study, Rendon et al. demonstrated significant improvement in Timed Up and Go and the ABC scale in the Wii-Fit group compared to a nonintervention control group that was only seen for assessments at baseline and six weeks after intervention [40]. Two RCTs that used Wii-Fit and had balance as outcome measures did not report between-group differences [43, 44].

Wii-Fit exercises are shown to improve motor function independent of balance. Two RCTs of Wii-Fit demonstrated statistically significant between-group differences on motor function in select populations of older adults. Saposnik et al. demonstrated significant improvement in the motor function in poststroke subjects in the Wii-Fit augmented standardized rehabilitation therapy group compared to the recreational therapy group [45]. Jorgensen et al. demonstrated significant improvement in leg muscle strength and no improvement in the secondary outcome of postural balance in physiotherapist supervised Wii-Fit group compared to the control group who were given shoe insoles to wear [46]. These two studies also had small sample sizes and had low dose of the intervention.

The current study adds to the findings of the prior studies by demonstrating that Wii-Fit produced a significantly greater effect on balance compared to the control intervention after controlling for baseline BBS scores. This is perhaps due to the differences in the dose of the intervention or other aspects of study design compared to previous studies. This includes analyzing the primary outcome measure of balance using intent-to-treat principles in a sensitivity analysis, adjusting for baseline, and having an attention control group. There were several other strengths of the study. First, 30% of the subjects were African Americans, which make the results more generalizable. Second, there was a high adherence to the program. In addition, none of the subjects stopped exercising before the time allotted for them to exercise; this suggests that making the program self-guided and tailored to comply with each subject's preferences and abilities was effective in keeping the subjects engaged. Third, we clearly demonstrated that subjects were able to safely participate in the Wii-Fit program with no study-related adverse events.

The current study has several limitations. Most notable one was the limited sample size. The study was a pilot investigation and was not a priori adequately powered to address all of the outcomes. The study population primarily consisted of male Veterans; although consistent with the older population enrolled within the Veterans Health Administration, this limits the generalizability. Another major limitation was the lack of blinding of the outcomes assessor. The assessor was not part of the exercise sessions; however, the assessor was aware of the group assignment. The use of Wii-Fit as an exercise modality was a limiting factor for morbidly obese subjects as the Wii balance board has an upper weight limit. Activity levels between exercise sessions were not recorded. Although there is no reason to believe that participants in either group were more active than others in between the sessions, such information could help explain the outcomes. Another limitation of the study design was the lack of postintervention follow-up to study the retention of benefits of exercise. The duration of the exercise program in our study was 8 weeks, shorter than the recommended duration of 12 weeks, which is reported to have the largest effect on balance [47]. The additional time may increase the power of the study to detect any significant differences in the secondary outcome measures. Additional studies will be needed to test the efficacy of Wii-Fit in a female population and on more sophisticated measures of balance such as center of pressure and ultimately on fall rates; a larger sample size will likely be required for such studies.

## 5. Conclusion

This study confirms the safety and feasibility of a Wii-Fit exercise program for community dwelling older adults with mild-to-moderate balance problems. Furthermore, it also demonstrates that Wii-Fit exergames can be used efficaciously in this population to improve balance.

## Figures and Tables

**Table 1:** Descriptive characteristics of exercise and control groups.

	All participants (*N* = 30)	Exercise group (*N* = 15)	Control group (*N* = 15)	*p *value^a^
Age, mean (SD)	68.0 (6.7)	67.5 (8.1)	69.0 (3.8)	0.71
Male, *n* (%)	26 (86.7)	13 (86.7)	13 (86.7)	>0.99
Race, *n* (%)				0.12
Non-Hispanic Caucasian	20 (66.7)	8 (53)	12 (80)	
Non-Hispanic African American	10 (33.3)	7 (47)	3 (20)	
Anthropometry				
Height (inches), mean (SD)	69.3 (3.6)	69.3 (3.8)	69.3 (3.4)	>0.99
Weight (lbs.), median (IQR)	206 (176, 236)	198 (173–262)	208 (176–227)	0.37
BMI (kg/m^2^), mean (SD)	30.6 (5.9)	32.0 (7.1)	29.3 (4.3)	0.21
Education, *n* (%)				>0.99
High school	6 (20.0)	3 (20.0)	3 (20.0)	
More than high school	24 (80.0)	12 (80.0)	12 (80.0)	
Number of comorbidities, median (IQR)	6.5 (4, 9)	7 (6–9)	5 (3–8)	0.27
Hypertension, *n* (%)	21 (70.0)	11 (73.3)	10 (66.7)	>0.99
Diabetes, *n* (%)	9 (30.0)	3 (20.0)	6 (40.0)	0.43
Heart disease, *n* (%)	3 (10.0)	1 (6.7)	2 (13.3)	>0.99
Hyperlipidemia, *n* (%)	21 (70.0)	11 (73.3)	10 (66.7)	>0.99
Depression, *n* (%)	11 (36.7)	4 (26.7)	7 (46.7)	0.45
Obesity, *n* (%)	14 (46.7)	8 (53.3)	6 (40)	0.72
Number of medications, median (IQR)	6.5 (4, 9)	8 (5–11)	6 (4–8)	0.32
Systolic blood pressure, mean (SD)	126.5 (9.6)	124.9 (8.9)	128.2 (10.3)	0.44
Diastolic blood pressure, mean (SD)	78.9 (4.5)	79.7 (3.6)	78.1 (5.2)	0.72
Pulse, mean (SD)	75.2 (9.3)	75.6 (9.2)	74.9 (9.7)	0.80

^a^Comparison of exercise and control groups using *t*-test or Wilcoxon rank sum test for continuous data or chi-square test for categorical data.

**Table 2:** Baseline outcome measures in exercise and control groups.

Variables	All Participants (*N* = 30) Mean (SD)	Exercise group (*N* = 15) Mean (SD)	Control group (*N* = 15) Mean (SD)	*p *value^a^
Berg Balance Scale (BBS)	49.3 (2.6)	48.5 (2.6)	50.1 (2.5)	0.08
Activities-Specific Balance Scale (ABC)	81.9 (14.6)	80.7 (12.1)	83.0 (17.1)	0.67
Modified Mini Mental State Exam (3MS)	94.5 (4.1)	94.1 (4.0)	94.9 (4.3)	0.60
Mini Mental State Exam (MMSE)	27.1 (1.7)	26.4 (1.6)	27.7 (1.7)	0.03^*∗*^
Short Form-36 (Mental)	52.8 (13.2)	56.8 (9.8)	48.8 (15.2)	0.10
Short Form-36 (Physical)	44.6 (8.8)	42.2 (8.3)	47.0 (8.9)	0.14
Short Form-36 (Physical function)	65.0 (21.1)	63.3 (24.5)	66.7 (17.8)	0.67

^a^Comparison of exercise and control groups using *t*-test or Wilcoxon rank sum test.

^*∗*^ *p* value significant at <0.05.

**Table 3:** Changes from baseline (4 or 8 weeks minus baseline) in outcomes for exercise and control groups and differences between the two groups.

	Change at 4 weeks Mean and 95% CI				Change at 8 weeks Mean and 95% CI			
	Exercise	Control	Difference^a^	*p *value^b^	Exercise	Control	Difference^a^	*p *value^b^
BBS	3.7 2.8 to 4.6	0.2 −0.6 to 0.9	3.6 2.3 to 4.8	<0.001^*∗*^	6.0 5.1 to 6.9	0.5 −0.3 to 1.3	5.5 4.3 to 6.7	<0.001^*∗*^
ABC	4.7 −1.0 to 10.4	−0.9 −6.1 to 4.2	5.6 −2.1 to 13.4	0.15	5.9 0.1 to 11.6	2.4 −2.8 to 7.5	3.5 −4.2 to 11.2	0.36
3MS	2.4 1.2 to 3.7	3.0 1.9 to 4.2	−0.6 −2.3 to 1.1	0.48	2.0 0.7 to 3.3	1.2 0.1 to 2.4	0.8 −0.9 to 2.5	0.36
MMSE	1.5 0.7 to 2.3	1.5 0.9 to 2.2	0.0 −1.1 to 1.0	0.95	1.1 0.3 to 1.8	0.8 0.1 to 1.5	0.3 −0.7 to 1.3	0.57
SF36:M	−0.2 −5.2 to 4.8	0.8 −3.6 to 5.3	−1.0 −7.8 to 5.7	0.75	4.2 −0.7 to 9.2	3.3 −1.2 to 7.7	1.0 −5.8 to 7.7	0.77
SF36:P	2.1 −1.8 to 5.9	1.0 −2.5 to 4.4	1.1 −4.2 to 6.4	0.67	0.9 −3.0 to 4.7	−0.1 −3.6 to 3.4	1.0 −4.3 to 6.2	0.71
SF36:PF	9.2 0.5 to 17.9	4.6 −3.2 to 12.4	4.6 −16.3 to 7.1	0.43	5.5 −3.2 to 14.2	3.3 −4.5 to 11.1	2.2 −13.9 to 9.5	0.70

^a^All means are estimates from repeated measures model of 4- and 8-week change from baseline. Difference reflects exercise group change minus control group change and is adjusted for corresponding baseline measure.

^b^ *p* values comparing exercise and control groups are model-based.

BBS: Berg Balance Scale; 3MS: Modified Mini Mental State Exam; MMSE: Mini Mental State Exam; ABC: Activities-Specific Balance Scale; PACES: Physical Activity Enjoyment Scale; SF36:M: Short Form-36 (Mental); SF36:P: Short Form-36 (Physical); SF36:PF: Short Form-36 (Physical function).

^*∗*^ *p* value significant at <0.05.

**Table 4:** Details of Wii-Fit exercises preferentially used by subjects in this study.

Category	Exercise	Details	Balance components targeted
Balance^a^	Ski Slalom	Maneuver between virtual poles while flexing knees and shifting weight	SB, DB, Coordination
	Bubble Balance	Lean forward, backward, left, right to guide a bubble down a course, avoiding obstacles	SB, DB, Coordination, Endurance
	Penguin Slide	Move a penguin on ice to pick a fish by moving center of gravity in mediolateral plane	SB, DB, Coordination
	Table Tilt	Directing balls into holes on a tilting platform by moving the center of gravity	SB, DB, Coordination, Endurance
	Soccer Heading	Strike a virtual soccer ball coming from the TV by moving the center of gravity	SB, DB, Coordination
	Tight Rope Walking	Rhythmic lateral weight shifting with outstretched arms to walk without falling	SB, DB, Coordination, Endurance

Yoga^a^	Deep Breathing	Inhale and exhale to video cues while maintaining steady posture	SB, DB, Endurance
	Half Moon	Lean upper body sideways with outstretched arms mirroring virtual instructor	SB, DB, Endurance, Strength
	Warrior	Stretch while bending one knee and outstretched arms	SB, DB, Endurance, Strength

Strength Training	Torso Twist^a^	Twist the trunk left and right with outstretched arms mirroring virtual instructor	SB, DB, Coordination, Strength
	Triceps Extension	Extend arm mirroring trainer with the controller in the hand	SB, Coordination, Strength

Aerobic	Basic Walk/Run	Walk in place while holding the controller and follow a cat on the screen	Warm up, DB, Coordination, Endurance

Training Plus^a^	Perfect 10	Mediolateral and anteroposterior movements to add numbers to make a score of 10	SB, DB, Coordination, Endurance
	Obstacle Course	Navigate obstacles while walking on the balance board and stopping at right moments	SB, DB, Coordination, Endurance, Strength
	Island cycling	Walk on the balance board to propel a cycle on the screen to collect flags on the route	SB, DB, Endurance
	Segway Circuit	Lean forward, backward, left, right to guide a Segway at hot air balloons to collect points	SB, DB, Coordination, Endurance

^a^Uses balance board to perform exercises.

DB = Dynamic Balance, SB = Static Balance.
